# Mechatronic Description of a Laser Autoguided Vehicle for Greenhouse Operations

**DOI:** 10.3390/s130100769

**Published:** 2013-01-08

**Authors:** Julián Sánchez-Hermosilla, Ramón González, Francisco Rodríguez, Julián G. Donaire

**Affiliations:** 1 Departamento de Ingeniería, Ctra. Sacramento s/n, E-04120. Universidad de Almería, Almería, Spain; E-Mail: jusanche@ual.es; 2 Departamento de Informática, Ctra. Sacramento s/n, E-04120, Universidad de Almería, Almería, Spain; E-Mails: frrodrig@ual.es (F.R.); jugarcia@ual.es (J.G.D.)

**Keywords:** agricultural robot, greenhouse, autonomous vehicle navigation, computer vision

## Abstract

This paper presents a novel approach for guiding mobile robots inside greenhouses demonstrated by promising preliminary physical experiments. It represents a comprehensive attempt to use the successful principles of AGVs (auto-guided vehicles) inside greenhouses, but avoiding the necessity of modifying the crop layout, and avoiding having to bury metallic pipes in the greenhouse floor. The designed vehicle can operate different tools, e.g., a spray system for applying plant-protection product, a lifting platform to reach the top part of the plants to perform pruning and harvesting tasks, and a trailer to transport fruits, plants, and crop waste. Regarding autonomous navigation, it follows the idea of AGVs, but now laser emitters are used to mark the desired route. The vehicle development is analyzed from a mechatronic standpoint (mechanics, electronics, and autonomous control).

## 1. Introduction

In recent years, greenhouse technologies have undergone many improvements, such as the better design of greenhouse structure, irrigation systems, fertilization, and climate-control systems. Nevertheless, many tasks in greenhouse crops are still performed manually, such as transplanting, harvesting, pruning, and the application of plant-protection products. In addition, the environmental conditions of typical greenhouses, with high temperatures and humidity, make work harsh and sometimes hazardous for the farm workers, especially in applying chemical products with little air renewal. In this context, different studies have examined the development of machinery for agricultural tasks in greenhouses. For example, the application of plant-protection products has advanced considerably in recent years, although spray guns continue to be extensively used. When spray guns are used the canopy is not sprayed uniformly and much of the spray is lost on the ground [1–4], and results in high chemical exposure of the operators [5,6]. Must be in numerical order Recently, as an alternative to using spray guns, spray equipment has been developed with vertical spray booms that increase the deposition in the canopy [7,8] and reduce the losses on the ground [3,4]. Some of these alternatives are self-propelled vehicles such as Fumimatic® (IDM S.L, Almería, Spain) and Tizona^®^ (Carretillas Amate S.L., Almería, Spain), or autonomous vehicles such as Fitorobot® (Universidad de Almería, Cadia S.L., Almería, Spain), designed specifically to move without difficulty over loose soils and in spaces with a large number of obstacles [9].

The design of proper navigation systems for autonomous vehicles moving in structured environments, such as greenhouses, constitutes a challenging research topic [10]. A promising approach, which has been successfully applied in open field crops as well as greenhouses, deals with automated guided vehicles or AGVs [11–14]. These vehicles rely on (inductive) sensors to follow metal pipes buried in the soil; typically, these metal pipes part of the greenhouse heating system. However, few projects have addressed the navigation problem of vehicles in greenhouses operating completely autonomously [15–18]. The main challenge of these systems is that localization approaches needed for feeding the closed-loop controllers would lead to inaccurate measurements after a few steps. For instance, absolute localization techniques, such as beacons, or relative localization approaches, such as odometry and dead-reckoning, would fail for long trajectories [19]. Even in the case of beacons the signal can be lost due to occlusions.

**Figure 1.**
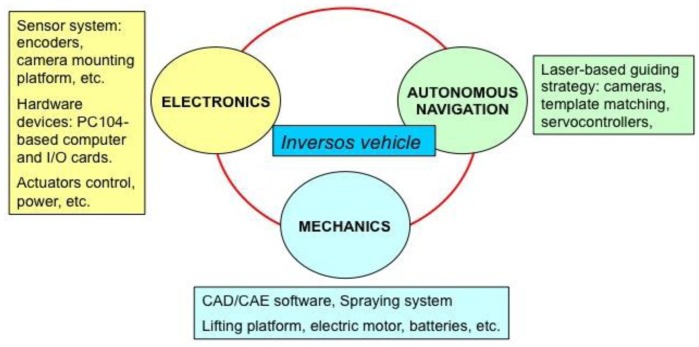
Mechatronic paradigm followed in this project.

The present work is part of a project to design and develop an electric mobile robot which moves through the rows of a greenhouse crop and performs tasks that are tedious and/or hazardous for human operators (as in the case of plant-protection product application), with safe, efficient, and economic results. This paper describes the entire project from a mechatronic (mechanics, electronics, and autonomous navigation) standpoint (Figure 1). Note that the work developed represents a comprehensive attempt to use the successful principles of AGVs (auto-guided vehicles) inside greenhouses, but avoiding the necessity of modifying the crop layout, and avoiding having to bury metallic pipes in the greenhouse floor.

This paper is organized as follows: a mechanical description of the developed autonomous multipurpose electric vehicle is provided in Section 2. The navigation strategy is discussed in Section 3. Section 4 deals with electronics and hardware components. Results showing the performance of the vehicle developed and the laser-based vision approach are detailed in Section 5. Finally, a discussion of the whole system is presented in Section 6.

## 2. Mechanical Description

The vehicle has been designed taking into account the following:

- It must move on a soft soil (typical of south-eastern Spanish greenhouses) and in an environment with many obstacles and tight spaces.
- It must have enough capacity for optimal work performance.
- It must perform different tasks within the greenhouse.

### 2.1. Dimensions and Frame

**Figure 2.**
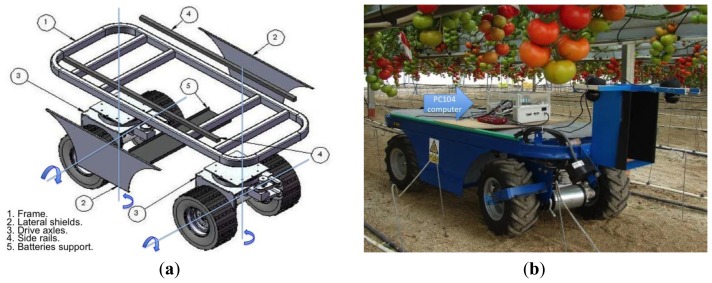
(**a**) CAD/CAE design of the vehicle. (**b**) Real vehicle moving in a greenhouse (tomato crop). Note the PC104-based computer onboard the vehicle and the vision cameras.

With the goal of satisfying the requirements listed above, a compact platform with four-wheel drive was developed (Figure 2). The dimensions of the vehicle are 0.71 m wide, 1.75 m long and 0.53 m height. This ensures accurate movement through the crop rows, which leaves a free path of approximately 0.8 m, and guarantees the possibility of turning in the greenhouse corridors, which are about 2 m wide.

The frame provides support for the batteries and all elements of the steering and traction system. As shown in Figure 2(a), the platform has a rail on each side; these rails serve to attach the different tools designed for spraying tasks, lifting platform, *etc.* Note that through this straightforward mechanism many tools can be easily used, diversifying the function of the original vehicle. Also, as shown in Figure 2, it has two lateral shields (2), made of steel plates to protect the batteries. The steering system and the propulsion system complete the vehicle. As the travel speed of the vehicle is low (maximum speed 1.5 m/s), a suspension system is not necessary.

### 2.2. Steering System

The mobile robot has no fixed axles. Both axles are pivoted for the steering system. This arrangement enables a tighter turning radius and more accurate tracking than 2-wheel steering models.

**Figure 3.**
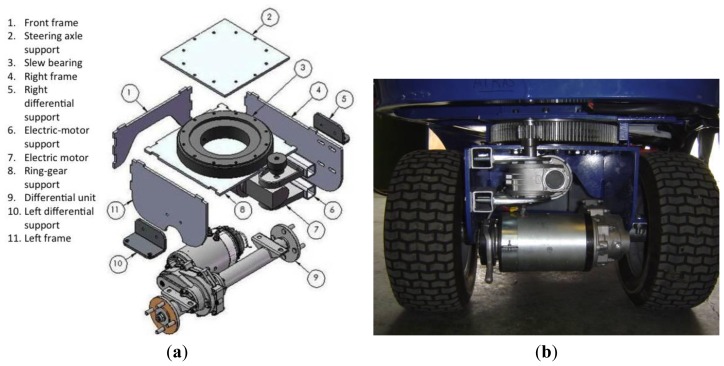
(**a**) CAD/CAE design of the steering axle. (**b**) Real image.

Each axle is attached to the vehicle frame with a slew bearing that is driven by a DC electric motor (Figure 3(a)). The slew bearing has 300 mm pitch diameter, 120 teeth, and module of 2.5 (model VE030A00, La Leonessa S.P.A, Carpenedolo, Brescia, Italy). The DC electric motor has 350 W (Edon motors S.L, Vallirana, Barcelona, Spain). An 18-tooth gear and module 2.5 is used to transfer the motor power to the slew bearing.

### 2.3. Propulsion System

The propulsion system needs to be easy to control, supply, and install. The maximum travel speed is fixed at 1.5 m/s to allow good control of the vehicle inside the greenhouse. Two drive axles driven by a S21-ME differential unit (Metalrota, S.r.l., Modena, Italy) are used. Each S21-ME differential unit (Figure 3) has a gear ratio of 32:1, an output torque of 400 Nm and is driven by a DC electric motors of 24 V and 900 W. Two traction batteries of 12 V and 170 Ah are used for the power supply. This calculation took into account the weight of the vehicle with the payload (about 500 kg), under the assumption that the vehicle is travelling on a soft soil surface at a slope of 7% and an acceleration of 0.38 m/s^2^. It also assumes that the sprayer pump is powered separately. It is important to remark that working with the vehicle under the maximum payload (500 kg), at maximum travel speed (1.5 m/s), and in continuous operation, using the 4-wheel-drive configuration, the batteries last 65 min and using the 2-wheel-drive configuration, the batteries last 130 min. However, if the vehicle moves in a non-continuous operation, it could operate for a longer time, even several days.

### 2.4. Tools for Agricultural Tasks

**Figure 4.**
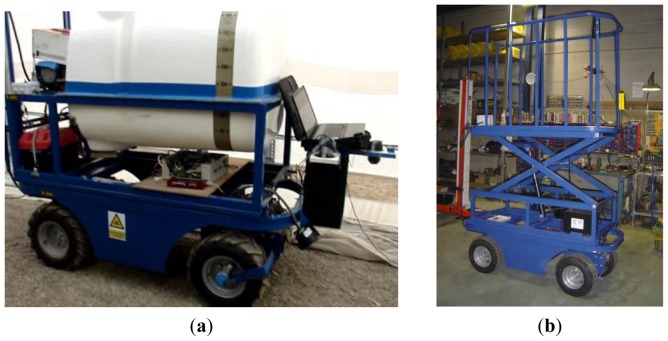
(**a**) Vehicle equipped with the spraying system (phytosanitary tank). (**b**) Vehicle equipped with the lifting platform (extended).

As mentioned above, the goal of this work is to design a multi-purpose vehicle for working inside greenhouses. Firstly, two tools were designed for the vehicle: a spraying system (Figure 4(a)) and a lifting system to reach high zones to perform tasks such as pruning and harvesting (Figure 4(b)).

The spraying system consists of a 400-litre tank, two vertical spray booms with four nozzles, an electrovalve of two positions that enable the activation or deactivation of the spraying, a proportional electrovalve to regulate the output pressure, and an electric pump. The lifting system, enabling tasks to be performed at 2.5 m above the ground, is made up of a platform of 1.75 × 0.71 m, two scissor arms which connect the platform to the base, and a hydraulic cylinder which extends the scissor arms. The hydraulic cylinder is activated by an electric hydraulic-pump module. The base of the lifting system is connected to the side rails of the vehicle.

## 3. Laser-Based Guiding Strategy

The purpose of the proposed strategy is to steer the autonomous vehicle following a sequence of laser emitters installed in a greenhouse, *i.e.*, the autonomous vehicle will follow a set of active laser emitters that will compose the desired route (these laser emitters were previously selected by a human operator).

**Figure 5.**
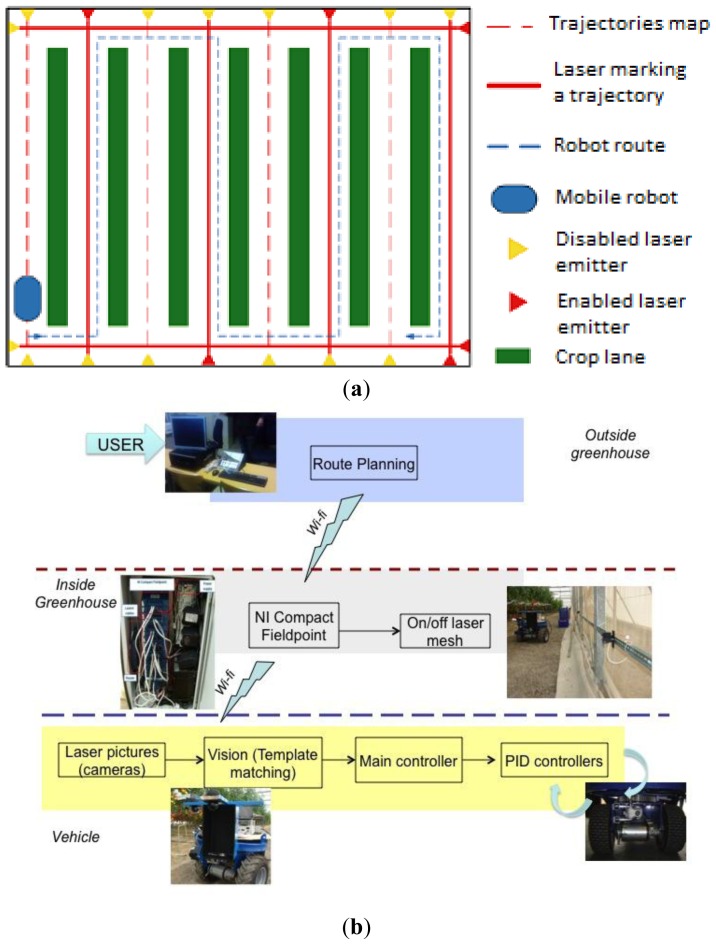
(**a**) Laser mesh in a typical Spanish greenhouse layout. (**b**) Elements composing the control architecture.

As shown in Figure 5(a), there is a set of laser emitters at the end of each corridor and two emitters in the transversal corridors of the greenhouse. Following this approach, many routes are available. At this point, two important remarks are necessary: (i) the proposed methodology functions mainly for straight-line corridors, although it could be adapted to other geometrical configurations (note that most greenhouses in southern Spain have a layout similar to the one shown in Figure 5(a), [8]; (ii) a fault-tolerant procedure runs on the autonomous vehicle that detects when there is a sudden stop of the laser ray hitting the front dartboard. Therefore when the laser beam is lost or false detections happen the vehicle stops until the laser beam is recovered. Note that such occlusions rarely occur in the scenario considered, since the laser emitters are located at the middle of the crop corridors where plants or leaves do not usually appear.

The navigation scheme, shown in Figure 5(b), is composed of two main layers, each running in different places and computers. First, there is a software application to manage the laser mesh in the greenhouse; this software runs in a computer located outside the greenhouse. All laser emitters are wired to a NI Compact FieldPoint® module which communicates with the computer. The user selects the route, *i.e.*, the sequence of laser emitters that will be activated, then these data are sent to the Compact Fieldpoint module. When the vehicle is moving communicates with the FieldPoint module, which activates the next laser emitters in the sequence. Note that in this way, human operator is needed only once (selection of reference path), and even better, no human operator is needed inside the greenhouse, which is very advisable for spraying tasks.

Finally a program deals with the vehicle control. It runs in the PC104-based computer on board the vehicle. A vision algorithm is used to detect the misalignment of the vehicle within a corridor and the end of a corridor. Note that the images are taken using a set of three cameras (Logitech Quickcam Sphere) pointing at three dartboards attached to the vehicle (see Section 4.3). The vision algorithm and the design of the dartboards constitute one of the main contributions of this work. Finally, there are two low-level Proportional Integral Derivative (PID) controllers or servocontrollers, which are devoted to control the wheels velocity and steering axles. It is important to point out that a human operator fixes the forward velocity (typically 0.5 m/s) and the rotation used for correcting the vehicle during misalignment-correction step.

### 3.1. Laser-Based Vision Approach

The laser-based vision approach is designed to process the image acquired from the front camera and an image from one of the two side cameras. Firstly, it has to detect the laser point (red point). Afterwards, it estimates the deviation of such point with respect to the dartboard centre that leads to estimating the vehicle deviation from the centre of the greenhouse corridor. The navigation control module sends the appropriate commands to the low-level PID controllers based on the detection of the laser: it moves forward while correcting the trajectory, or turns into a new corridor. When an offset is detected within a straight corridor, the procedure implemented consists of making small turns until the offset is corrected.

For detection of the laser point, two approaches have been tested. Firstly, an RGB-filter-based approach was tested [20]. The main drawback of that approach is that it looked for the laser point within a static fixed threshold in the RGB colour histogram of the image. Then if laser point was not inside this range it was not detected. Secondly, a template-matching approach was tested. This method is defined as the process of locating the position of a sub-image inside a larger image. The sub-image is called the *template* and the larger image is called the *search area* [21,22]. This process involves shifting the template over the search area and computing the similarity between the template and a window in the search area. There are several methods to address the template matching. In this case, the cross-correlation solution has been implemented. It is based on calculating an array of dimensionless coefficients for every image position (s,v):

(1) $$R(s,\nu )=\sum_{i=0}^{h-1}\;\sum_{j=0}^{\rho -1}\left(T(i,j)-\bar{T}(i,j))(I(i+s,j+\nu )-\bar{I}(i+s,j+\nu )\right),$$

where *h* and ρ are the height and the width of the template, respectively, *T(i,j)* and *I(i,j)* are the pixel values at location (*i*,*j*) of the template and the current search area, respectively, and *T̄*,*Ī* are the mean values of the template and current search area, respectively.

The software was programmed in C language using the open source computer vision library “OpenCV” (version 1.1) [23]. In this case, the OpenCV function for template matching *cvMatchTemplate* was used.

### 3.2. Laser-Based Vision Approach. Practical Considerations

Before the physical experiments were conducted to check the performance of the template-matching approach, some important issues were addressed. First, the image size was analyzed in order to achieve an appropriate computation load and a good success during the matching. After checking several standard sizes such as 1,048 × 786 pixels, 800 × 600 pixels, 640 × 480 pixels and 320 × 240 pixels, the size 640 × 480 pixels, was considered the most appropriate as a compromise between low computational burden and low false matches. Then, the template size was also checked in order to minimize false matches. Through physical experiments, the size of 51 × 51 pixels was selected. The laser-based vision approach achieves a computation time less than 0.15 s using the PC104-based computer detailed in Section 4.

On the other hand, under some greenhouse lighting conditions, the images were too bright, leading to false matches, since the laser point could not be distinguished from the background. As a means to avoid this undesired effect, an image with no direct sun light condition was subtracted from each new image acquired. This simple step darkens bright images, reducing the probability of false matches.

## 4. Electronics and Hardware Components

### 4.1. Sensors

**Figure 6.**
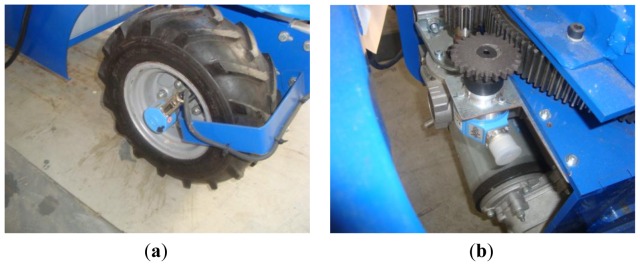
(**a**) Incremental encoder used to measure the angular velocity of the wheel (linear velocity of the vehicle). (**b**) Absolute encoder used to measure the angular position of the steering axle. Note the mechanical coupling device.

Four sensors were installed in the vehicle in order to control the wheels velocity and the pivotal steering axle orientation (low-level PID controllers). In particular, two incremental optical encoders (DRS 61, Sick, Waldkirch, Germany) were attached to the rotation axle of the front right and rear left wheels to measure the angular velocity (Figure 6(a)). Furthermore, two absolute optical encoders (ARS 60, Sick) were attached to the steering axles to measure the orientation (Figure 6(b)). The main features of these encoders are summarized in Table 1.

### 4.2. Computer on Board the Autonomous Vehicle

The autonomous vehicle is controlled by an embedded system based on the PC104 standard [24] (PCM-3362N, Advantech, Irvine, CA, USA). This system is based on the Industrial Standard Architecture (ISA)-bus specifications to work in embedded devices. The main reasons why the PC104-based computer was selected are: reduced dimensions, low power supply and ease of integration of new input/output cards. The main characteristics of this PC104-based computer are summarized in Table 2. Figure 2(b) shows the PC104-based computer on board the vehicle.

Some input/output digital/analogue boards have been connected to the PC104 bus, so that the signals of the different sensors onboard the vehicle can be read and commands can be sent to the actuators of the vehicle. A counter board (PCM-3780, Advantech) with digital input deals with encoders; one analogue outputs board (DM8520HR, RTD, State College, PA, USA) are used for the wheel motors and spraying valves.

### 4.3. Camera-Support Mechanism

**Figure 7.**
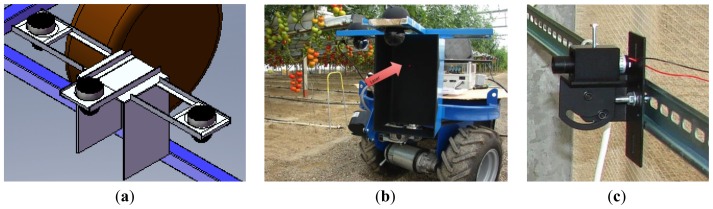
(**a**,**b**) Camera mounting platform in the front part of the vehicle. [20] Note the red laser point in the front dartboard. (**c**) Laser emitter installed in a greenhouse.

Regarding the proposed laser-based vision-guiding strategy, a fundamental issue is to design a mounting platform for the cameras and dartboards in the front part of the vehicle. This mechanism is composed of one front panel and two lateral panels. Each panel has one camera pointing at it (Figure 7). The front camera takes pictures when the robot moves along a straight corridor. The two lateral cameras take pictures when the robot arrives at the end of a corridor and is ready to turn to the left or to the right. This direction is selected depending on which of the two cameras detects the laser point. It bears mentioning that the mounting platform is firmly attached to the robot frame, ensuring low vibration. Furthermore, this mounting platform protects the cameras from crashes with unexpected obstacles. The vehicle dartboards are large enough to capture the incidence of the laser. In particular, it has a front width of 0.19 m, which is the difference between the corridor width and the vehicle width. On the other hand, a matte-black material covers the interior structure in order to avoid light reflections.

### 4.4. Laser Emitters

As discussed, laser emitters constitute a key element of the guiding strategy proposed in this work. In order to determine the proper laser emitter, physical experiments were conducted in a greenhouse environment. At the end, a red laser emitter (LTG, Apinex, Montreal, Canada) was selected (Figure 7(b)). Its main features are a low-cost price, maximum output power of 5 mW, which is a balance between safety standards (UNE-EN 60825-1/A-11) and the visibility at long distances and adjustable focal capacity to concentrate the beam for long distances.

## 5. Results

### 5.1. Vision Results

In order to check the performance of the vision approach, we conducted many experiments under different light conditions and with different laser-point diameters. In particular, the most informative experiments were conducted under direct sunlight conditions, diffuse light conditions (greenhouse environment), and non-direct light conditions (typical light conditions inside a warehouse). Different laser-point diameters were tested because, when the autonomous vehicle is far from the laser emitter, the laser point will be wider than when the autonomous vehicle is closer to the laser emitter.

**Figure 8.**
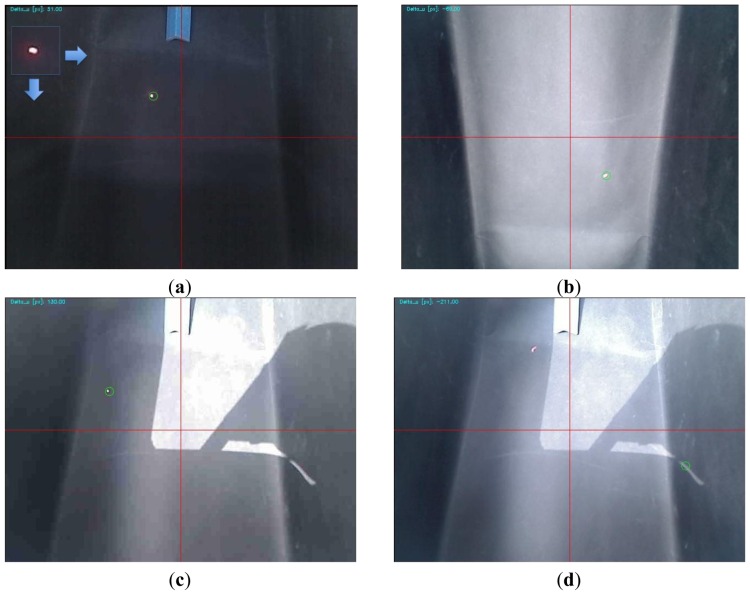
Results of some physical experiments testing the performance of the template matching approach. (**a**) Closed environment (similar light conditions to a warehouse). Note the template used at the top left part of the figure. (**b**) Greenhouse (diffuse light conditions). (**c**) Outdoor environment with direct sunlight conditions. Note the success of the matching detecting a very small laser point. (**d**) Outdoor environment with direct sun light (false matching).

For a summary of the physical experiments, see Figure 8. For explanatory purposes, we added a grid to these images to emphasize the misalignment of the vehicle with respect to the dartboard centre (y-axis). Furthermore, the green circle represents the position of the point with the maximum value after the matching, *i.e.*, the point where the template was positioned. First, Figure 8(a) deals with a picture taken in a closed environment with no direct light. Under these conditions the success of the matching was 99.13% (experiment carried out with 115 images with one false match). Figure 8(b) shows a picture taken in a greenhouse (conditions under which the robot is designed to work). In this case the success of the matching process was also very high, although some false matches were found with very bright images (experiment performed using 103 images with 10 false matches, 90.29%). Finally, two pictures are shown from an experiment made under direct sunlight conditions (Figure 8(c,d)). It is important to highlight that these were not the typical light conditions under which the autonomous vehicle is meant to operate. However, the success rate is close to 70% when the laser point does not strike within the brightest region of the picture, in this case, the top left corner.

### 5.2. Autonomous Operation. Preliminary Results

**Figure 9.**
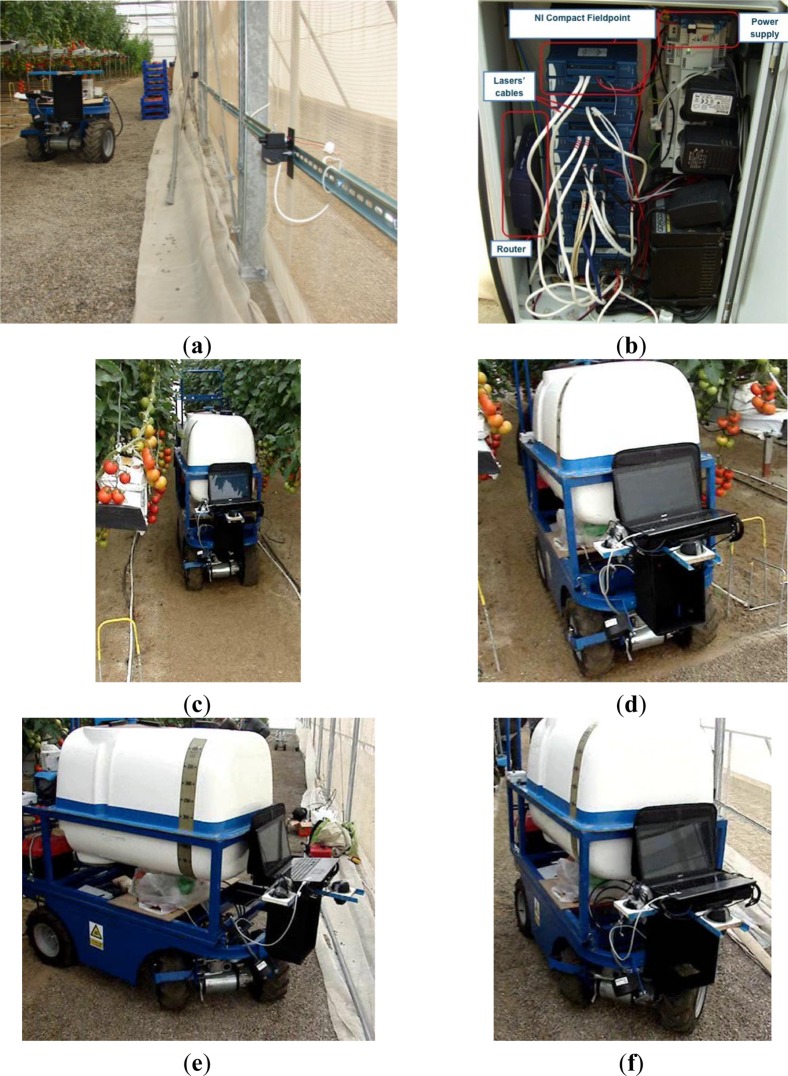
Results of some physical experiments testing the performance of the template-matching approach. (**a**) Laser emitters. (**b**) NI Compact Field Point. (**c**) Vehicle in the greenhouse corridor. (**d**) Leaving the corridor. (**e**) Turning to the right side. (**f**) Forward motion in the new corridor.

Before autonomous operation of the vehicle was tested, the set of laser emitters was installed in a greenhouse. As depicted in Figure 9(a), laser emitters were attached to the greenhouse walls using DIN® rails, permitting easy manipulation to adapt to changes in the crop layout. Then each laser emitter was wired to the NI Compact FieldPoint® module (Figure 9(b)).

**Figure 10.**
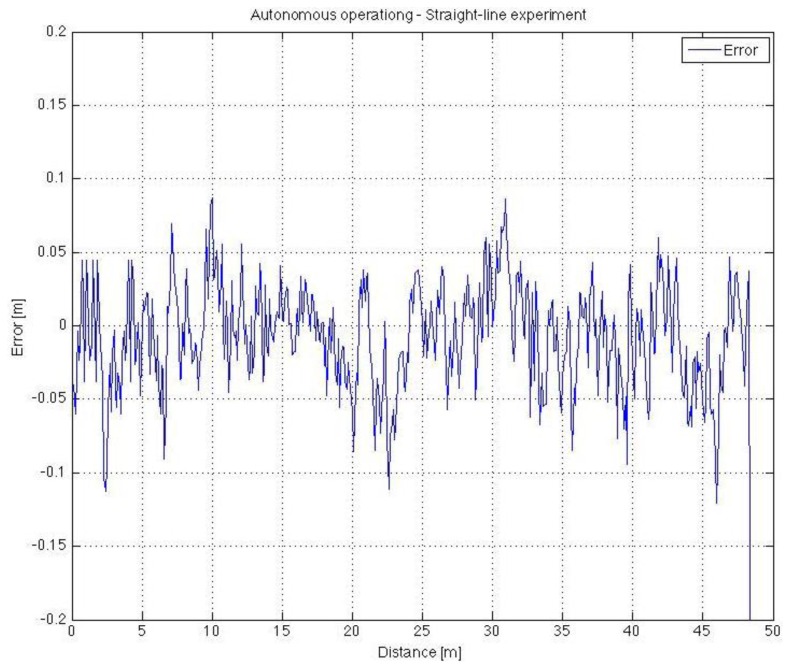
Physical experiment.

After the installation and testing of the laser emitters and of the NI Compact FieldPoint® module, several tests of the vehicle were made under autonomous operation conditions. Figure 9(c) shows the vehicle moving autonomously in a greenhouse (corridor length 50 m). Videos on the automatic operation of the vehicle can be seen in the following link: http://aer.ual.es/ramon/cta/videosCTA.htm. Note that for these preliminary experiments a laptop was used for controlling the vehicle (in the future the PC104-based computer will be employed instead). Figure 10 shows the deviation between the vehicle and the centre of the front dartboard, which must be aligned with the centre of the corridor (the laser emitter is just in the middle of the corridor). In this case, the vehicle follows properly the greenhouse corridor with very small corrections (mean = −0.09 m, mode = −0.04 m). Note that the corridors of this greenhouse were 1 m wide, then the maximum deviation of 0.1 m does not lead to any crop damage (recall that the vehicle is 0.71 m wide). It is important to point out that low-level controllers (PID) were not properly tuned, for that reason the vehicle oscillates along straight path. Future work will be devoted to adjust again these PID controllers.

The main conclusions drawn from these tests were: (i) the guided system functions correctly under different light conditions; (ii) the guided system functions correctly in the turns when the vehicle leaves one corridor to enter another, although situations were detected in which narrow corridors forced the vehicle had to manoeuvre more in order to centre itself in those corridors; (iii) wheel spinning situations could arise, although this situation was reduced when the vehicle was equipped with an implement such as the scissor lift or the spray system.

## 6. Discussion and Conclusions

An autonomous multipurpose electric vehicle was designed and developed for agricultural tasks in greenhouse crops, using CAD/CAE software. This small-sized vehicle can move with great manoeuvrability in an area with many obstacles, tight spaces, and can work with different tools to performer several tasks such as spraying, pruning, *etc.* An important conclusion from physical experiments regarding mechanical design was the tyre used for the wheels. Initially a flat-profile tyre was used (see Figure 3(b)), but the vehicle had a high difficulty in movement because greenhouses have loose sandy soil that produces a noticeable slip. For this reason this type was replaced by a lug-profile tyre (see Figure 2(b)). This tyre makes possible successful movement inside the greenhouse.

This vehicle is different from the successful project Fitorobot developed previously at the University of Almeria [15]. Being an electric vehicle, it therefore eliminates Fitorobot's disadvantages such as the emission of gases inside the greenhouse, which can be toxic to humans, and it minimizes noise. It also has wheels that reduce soil damage caused by the Fitorobot tracks.

Regarding the laser-based vision strategy for guiding purposes, it follows the principles of auto-guided vehicles but with the advantage that it does not require a costly and a tedious modification of the greenhouse. In this sense, this design is meant to be a practical contribution to agriculture rather than a theoretical contribution to robotics community. The vision algorithm based on template matching provides proper performance according to the light conditions under which the real autonomous vehicle would operate. Note that before trying the proposed laser-based navigation strategy, several well-known navigation strategies were evaluated. Firstly, the possibility of using a typical AGV was unfeasible since at the Southeast of Spain hardly any greenhouses use buried metallic pipes for heating. Secondly, different solutions based on satellite signal (GPS) and radio communication were analyzed, but due to the plastic and metallic structure of a greenhouse the signal was lost quite frequently. Finally, solutions based on RFID or landmarks were discarded due to plant and metallic pillar occlusions. It is also important to highlight that the installation costs of the laser system are much smaller than some of these other strategies. For instance, in our case, the laser emitters installed cost 20€; the greenhouse used for experiments had 25 corridors and two longer corridors, then 27 laser emitters were needed, leading to a total cost of less than 555€.

Currently the safety procedure consists of stopping the vehicle when no laser point is detected or when false matches happen. Furthermore, a manual remote controller is used for fully stopping the vehicle in case of collisions. In this sense, future works will focus on mounting an ultrasonic sensor and several bumpers around the vehicle. The work proposed in this paper is under patent (Ref. P201101119).

## Figures and Tables

**Table 1.** Features of the installed sensors.

**Sensor**	**Mark, Model**	**Range**	**Resolution**	**Signal Output**
Incremental encoder	Sick, DRS61	0–360°	1,024 pulse/rev.	Pulse
**Absolute Encoder**	**Sick, ARS60**	**0–360°**	**-**	**Gray code**

**Table 2.** Main features of the embedded system.

**Feature**	**Description**
CPU	Intel Atom N450 1.67 GHz
Memory	SDRAM 2 GB
Cache	512 KB
Chipset	Intel Atom N450
Interfaces	4 USB, Ethernet, Audio, Serial, PS2.
Graphic Card	Intel Gen 3.5 DX9
Power	12 VDC
Temperature	0–60°
**HDD**	**160 GB (S-ATA)**
